# Redescription of types of three species of Leptonetidae Simon, 1890 from China (Arachnida, Araneae)

**DOI:** 10.3897/zookeys.1000.57660

**Published:** 2020-12-03

**Authors:** Jinxin Liu, Zongguang Huang, Xiang Xu, Haiqiang Yin

**Affiliations:** 1 College of Life Science, Hunan Normal University, Changsha 410081, Hunan, China Hunan Normal University Changsha China; 2 The National & Local Joint Engineering Laboratory of Animal Peptide Drug Development (Hunan Normal University), National Development and Reform Commission, Changsha, Hunan 410081, China Hunan Normal University Changsha China

**Keywords:** *
Leptoneta
*, *
Leptonetela
*, new combination, taxonomy

## Abstract

Three species of the genus *Leptoneta* Simon, 1872 deposited at Hunan Normal University, Changsha, China, are examined and redescribed. Two species are transferred from *Leptoneta* Simon, 1872 to *Leptonetela* Kratochvíl, 1978, and the following new combinations are proposed: *Leptonetela trispinosa* (Yin, Wang & Wang, 1984), **comb. nov.** (♀♂), and *Leptonetela unispinosa* (Yin, Wang & Wang, 1984), **comb. nov.** (♂). The generic placement of *Leptoneta monodactyla* Yin, Wang & Wang, 1984 is maintained. Detailed descriptions, illustrations, and a distribution map for all three species are provided.

## Introduction

Leptonetids are small in size, usually less than 3 mm, with the body color entirely pale or yellowish (sometimes color varying between pale and yellowish) (Lin and Li 2010; Le Peru 2011). *Leptoneta* Simon, 1872, the type genus, comprises 68 species and is the second largest genus in the family (the genus *Leptonetela* Kratochvíl, 1978 is the largest with 108 species) (WSC 2020). The first *Leptoneta* species reported from China was *Leptoneta huanglongensis* Chen, Zhang & Song, 1982, which was collected from a cave. To date, 22 *Leptoneta* species have been described from China (Chen et al. 1982, 1984, 1986, 2000, 2010; Yin et al. 1984, 2012; Song and Xu 1986; Song and Kim 1991; Chen and Zhang 1993; Zhu and Tso 2002; Chen and Zhu 2008; Tong and Li 2008; WSC 2020). We reexamined all of the type specimens deposited in Hunan Normal University which were originally described as members of *Leptoneta*, including *Leptoneta monodactyla* Yin, Wang & Wang, 1984, *Leptoneta trispinosa* Yin, Wang & Wang, 1984, and *Leptoneta unispinosa* Yin, Wang & Wang, 1984. Males of *Leptoneta trispinosa* and *Leptoneta unispinosa* have characteristics of the genus *Leptonetela*, including strong palpal femoral spines absent (Figs 6A, 8C) and large palpal tibial spurs present (Figs 6B, 8D) (Lin and Li 2010; Wang and Li 2011). In this work, we transfer both species to the genus *Leptonetela*: *Leptonetela trispinosa* (Yin, Wang & Wang, 1984) comb. nov. and *Leptonetela unispinosa* (Yin, Wang & Wang, 1984) comb. nov. The number of the known *Leptoneta* and *Leptonetela* species from China changes from 20 and 98, respectively, to 22 and 96.

## Materials and methods

All specimens examined in this study are deposited in the College of Life Sciences, Hunan Normal University (**HNU**). Specimens were examined using an Olympus SZX16 stereomicroscope and an Olympus BX53 compound microscope. Photographs were taken with a Canon PowerShot G12 digital camera mounted on an Olympus BX53 compound microscope. Female genitalia were cleaned with lactic acid before being photographed. Both the male palp and female genitalia were examined, photographed, and illustrated after dissection. The data in original description was kept unaltered. Eye diameters were taken at the widest point. Leg measurements are given as total length (femur, patella, tibia, metatarsus, tarsus). Leg segments were measured on their dorsal sides. All measurements are in millimeters (mm). The left palpi and chelicerae of male spiders are illustrated, except where otherwise indicated.

Terminology in the present paper follows Wang et al. (2017) and He et al. (2019). The abbreviations used in the text and figures are as follows:

## Taxonomy

### Family Leptonetidae Simon, 1890

#### Genus Leptoneta

Taxon classificationAnimaliaAraneaeLeptonetidae

Simon, 1872

C99CA8DF-6794-566D-8ADD-FDD9FF9C2B32

##### Type species.

*Leptoneta convexa* Simon, 1872.

##### Type locality.

Ariége, France.

#### Leptoneta monodactyla

Taxon classificationAnimaliaAraneaeLeptonetidae

Yin, Wang & Wang, 1984

8877ACC2-1CBC-54FB-AE21-F3BAF00DAA78

Figures 1 , 2 , 3

Leptoneta monodactyla Yin et al., 1984: 366, fig. 2a–d (♂); Song 1987: 104, fig. 67 (♂); Song, Zhu and Chen 1999: 51, fig. 21H, I (♂, reproduction of the original figure); Yin et al. 2012: 156, fig. 26a–d (♂).

##### Material examined.

***Holotype*** ♂ (HNU, Lept-*Leptoneta*-0001-001): **China**, Hunan Province, Lingxian County, 5.XII.1982, leg. Jiafu Wang (information on the label of the type) [Lingxian is an old place name and now belongs to Hengyang City. The detailed information of the locality: Hunan Province, Hengyang City, Linghu Village (113°42'N, 26°30'E)].

##### Diagnosis.

**Figure 1.**
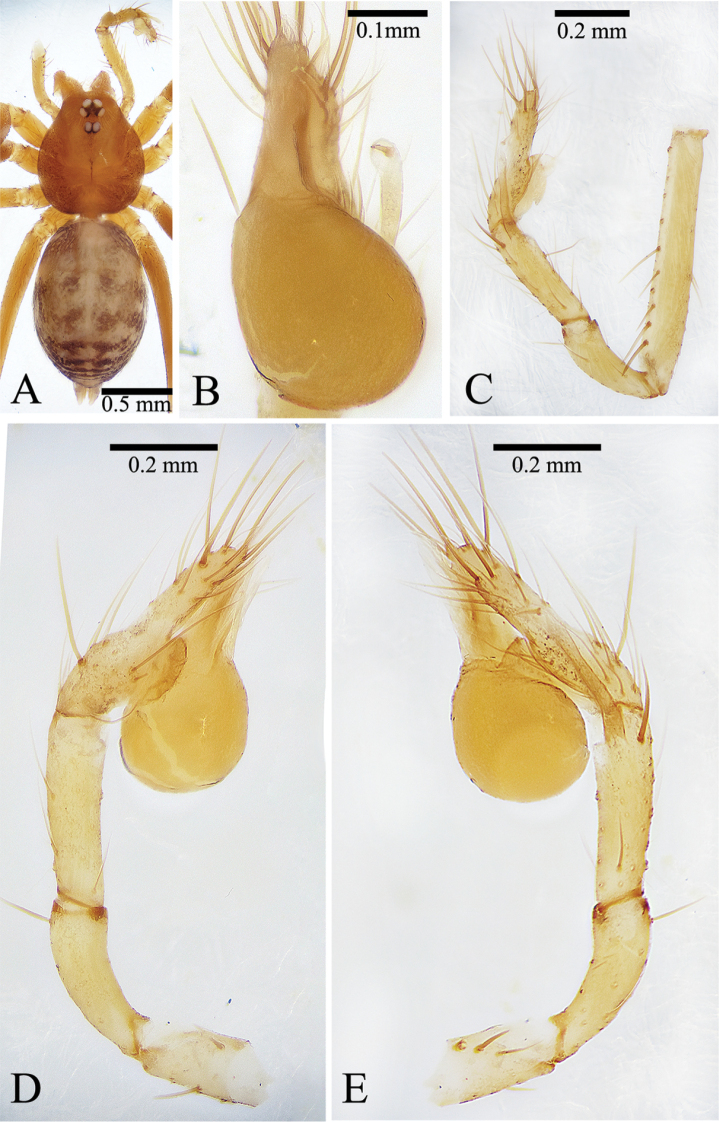
*Leptoneta monodactyla*Yin et al., 1984, holotype male **A** habitus, dorsal view **B** palpal bulb, ventral view **C** right palp (show the whole situation from patella to tarsus, but palpal bulb is missing), retrolateral view **D** palp, prolateral view **E** palp, retrolateral view.

**Figure 2.**
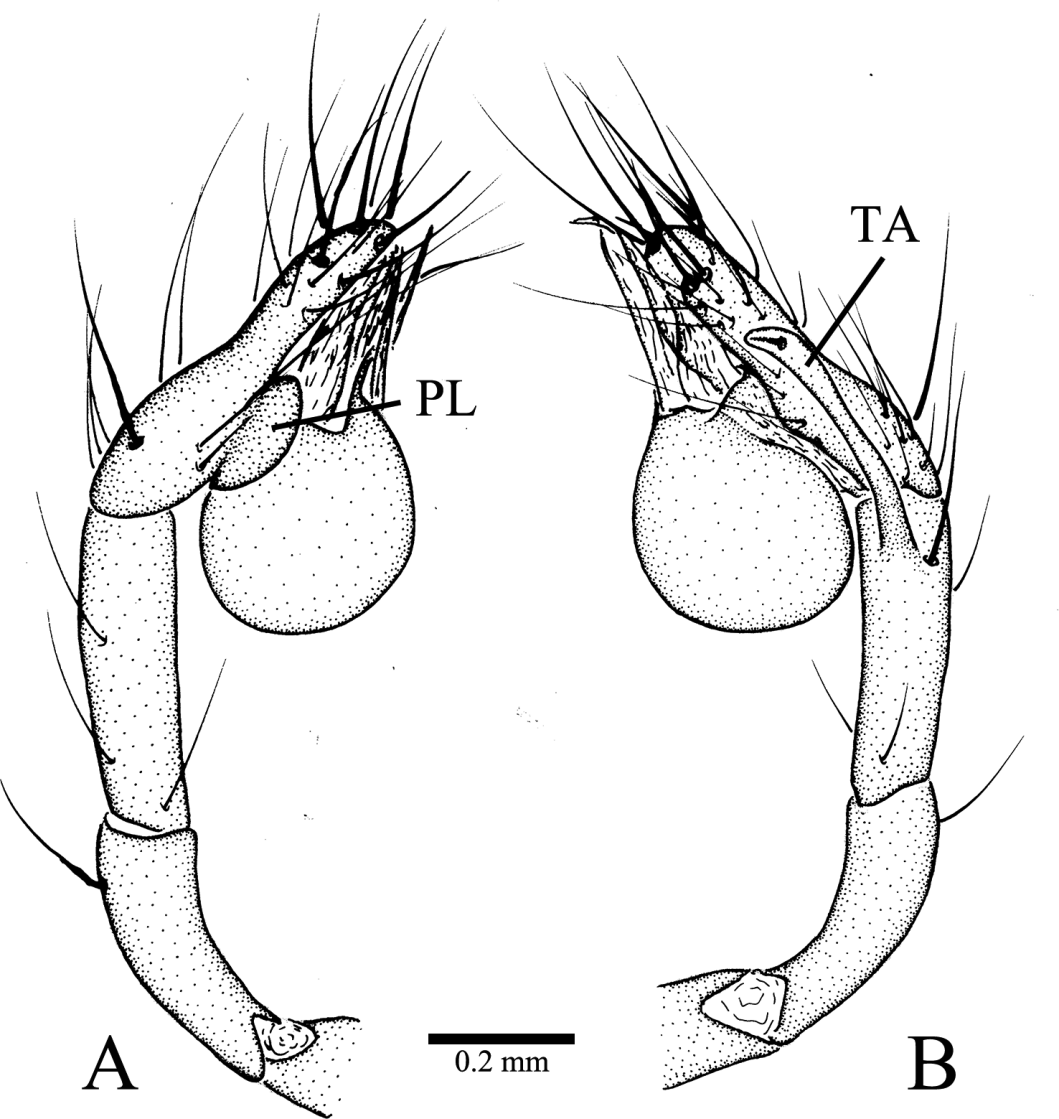
*Leptoneta monodactyla*Yin et al., 1984, Palp of holotype male **A** prolateral view **B** retrolateral view. Abbreviations: PL, prolateral lobe; TA, tibial apophysis.

**Figure 3.**
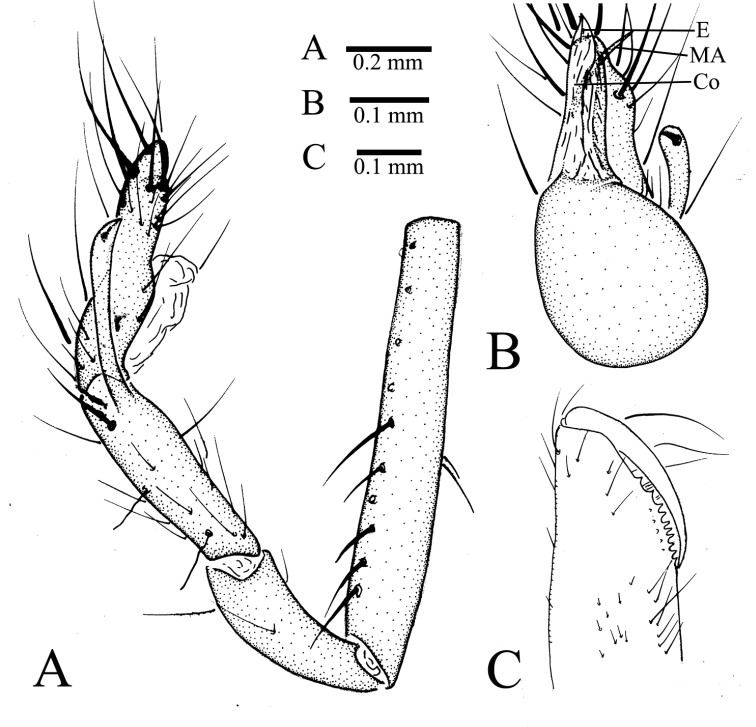
*Leptoneta monodactyla*Yin et al., 1984, holotype male **A** right palp (show the whole situation from patella to tarsus, but palpal bulb is missing), retrolateral view **B** palpal blub, ventral view **C** right chelicera (because teeth of left chelicera are broken), retrolateral view. Abbreviations: Co, conductor; E, embolus; MA, median apophysis.

Male is similar to that of *Leptoneta huanglongensis*Chen et al., 1982 in having a long tibial apophysis (TA) of palp, but differs by the detailed characters of TA (claviform, gradually more transparent from the base to the tip, with a small spine on its tip in this species vs finger-shaped and bifurcate distally in *L. huanglongensis*) (compare Figs 1B, C, E, 2B, 3A, B with Chen et al. 1982: fig. 3).

##### Description.

***Holotype*** Male. Body (Fig. 1A) length 2.20, carapace 0.85 long, 0.70 wide, abdomen 1.35 long, 0.75 wide (data from original description by Yin et al. 1984: 366). Carapace brown. Six eyes, ALE and PLE connected to each other by their black bases, PME separated from ALE and PLE. Thoracic median groove deep brown, needle-shaped. Cervical grooves and radial furrows deep brown, indistinct. Chelicerae yellowish brown, with ten promarginal (teeth gradually becoming smaller and denser from distal end to base of chelicera) and five retromarginal teeth (Fig. 3C). Endites deep brown. Labium deep brown, fused to sternum. Sternum deep brown, peltate. Legs deep yellow; measurements: I 8.20 (2.15, 0.30, 2.50, 2.00, 1.25); II 5.40 (1.50, 0.25, 1.60, 1.30, 0.75); III 4.35 (1.25, 0.25, 1.20, 1.10, 0.55); IV 6.60 (1.75, 0.25, 2.10, 1.60, 0.90) (data from original description by Yin et al. 1984: 367). Abdomen brown, ovoid, with wide, horizontal wave stripes (Fig. 1A).

Male palp as illustrated in Figs 1B–E, 2A, B, 3A, B. Femur with 10 ventral spines and five dorsal spines (Fig. 3A). Patella with several irregularly arranged setae besides distinct dorsal spine (Fig. 1D, E). Tibia with two trichobothria dorsally (Fig. 3A), with distal special apophysis (TA) and distal spine retrolaterally. TA clavate, gradually more transparent from base to tip, with small spine on its tip (Fig. 2B). Tarsus slightly sunken and contracted at middle position, with one distal long spine, three long dorsal spines, two long retrolateral, and two long prolateral spines on distal half, and one long dorsal spine on basal half (Figs 1D, E, 2A, B). Palpal bulb oval, with smooth surface. Conductor membranous, long, upright. Embolus smooth and small, similar color as conductor. Median apophysis needle-shaped, starting at anterior margin of palpal bulb prolaterally (Figs 1B, 3B). Prolateral lobe (PL) medium-sized, elliptical (Figs 1D, 2A). Cymbium not branched distally (Figs 1A–C, 2A, B, 3A).

**Female.** Unknown.

##### Distribution.

Only known from the type locality, Hunan, China (Fig. 9).

##### Remarks.

According to Platnick (1986, 2007) and Le Peru (2011), all *Leptoneta* species are limited to the western Mediterranean region and all those from outside the Mediterranean region are probably misplaced. Also, as stated by Tong and Li (2008), the Chinese *Leptoneta* species should probably be included in one or more new genera. The original designation of *Leptoneta monodactyla*Yin et al., 1984 is retained in this work pending comprehensive revisionary work.

#### Genus Leptonetela

Taxon classificationAnimaliaAraneaeLeptonetidae

Kratochvíl, 1978

1124EBB2-FA00-577E-8B6F-2EC28C8289A4

##### Type species.

*Sulcia kanellisi* (Deeleman-Reinhold, 1971).

##### Type locality.

Koutouki Cave near Ljopessi, Greece.

#### Leptonetela trispinosa

Taxon classificationAnimaliaAraneaeLeptonetidae

(Yin, Wang & Wang, 1984) comb. nov.

01C43FD9-5A2C-55A0-8606-B51C22AD62FE

Figures 4 , 5 , 6

Leptoneta trispinosa Yin et al. 1984: 364, fig. 1a–f (♂♀); Song 1987: 105, f. 68 (♂♀); Song et al. 1999: 51, figs 20R, 21L–M (♂♀, reproduction of the original figure); Yin et al. 2012: 157, fig. 27a–f (♂♀).

##### Material examined.

***Holotype*** ♂ (HNU, Lept-*Leptonetela*-0001-001): **China**, Hunan Province, Changsha City, Mountain Yuelu, 25.V.1982, Jiafu Wang leg.; ***paratypes*** 3♂3♀ (HNU, Lept-*Leptonetela*-0001-002–007), same data as holotype (information on the label of the type) [Mountain Yuelu: 112°58'N, 28°12'E].

##### Diagnosis.

**Figure 4.**
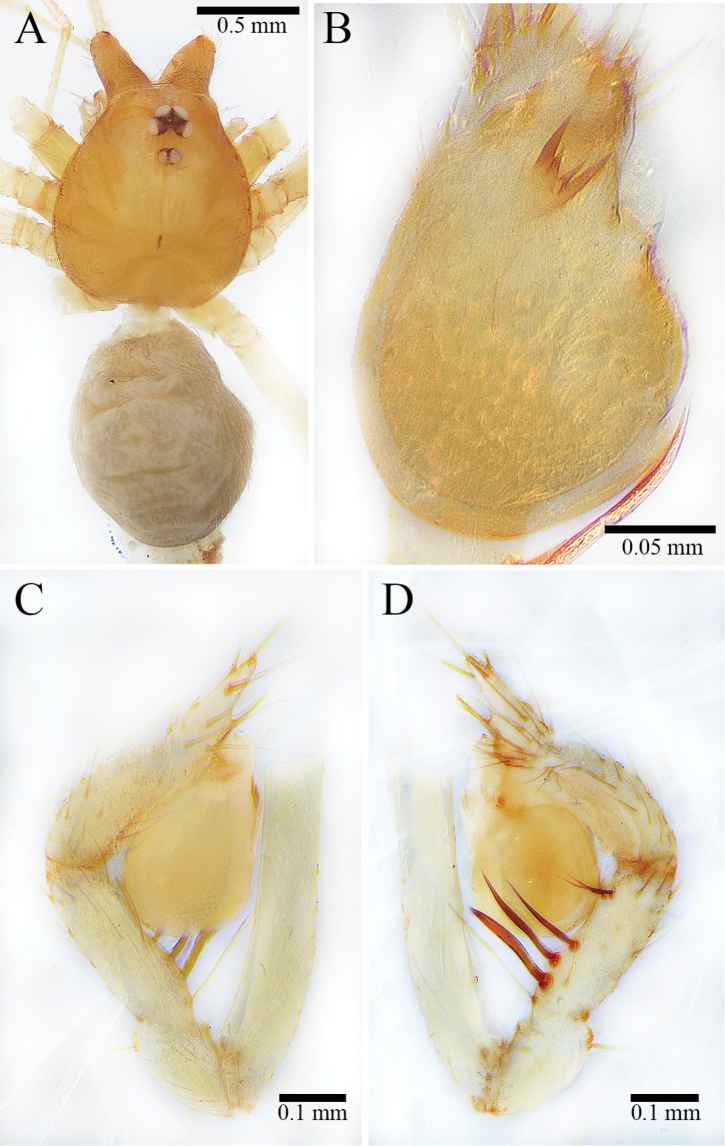
*Leptonetela trispinosa* (Yin et al., 1984), comb. nov., holotype male **A** habitus, dorsal view **B** palpal bulb, ventral view **C** palp, prolateral view **D** palp, retrolateral view.

**Figure 5.**
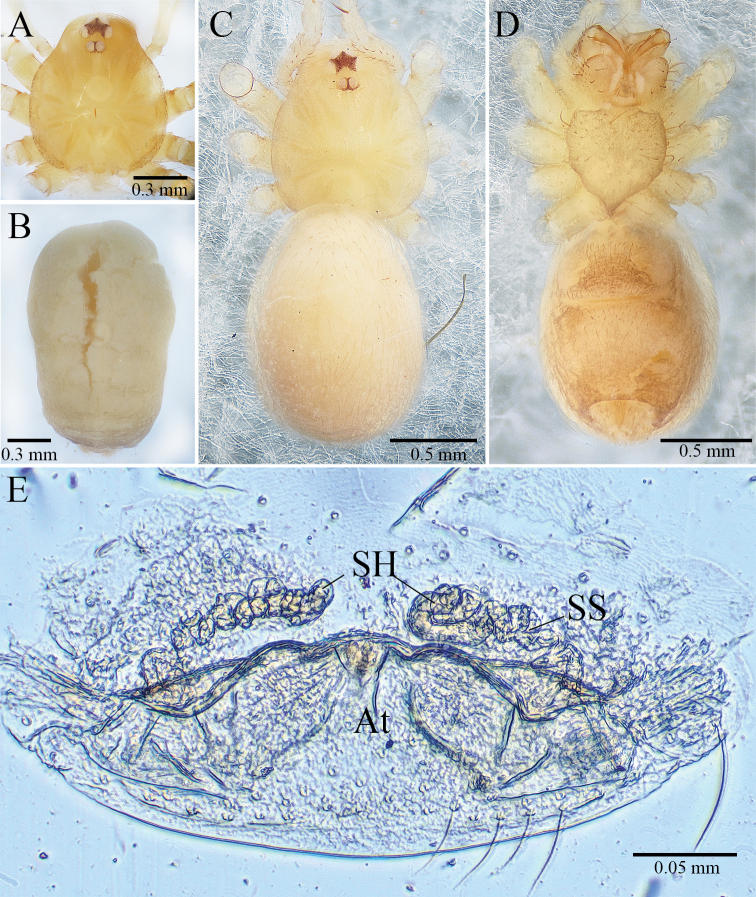
*Leptonetela trispinosa* (Yin et al., 1984), comb. nov., paratype female **A** carapace, dorsal view **B** abdomen, dorsal view **C** habitus, dorsal view **D** habitus, ventral view **E** vulva, dorsal view. Abbreviations: At, atrium; SS, spermathecae stalk; SH, spermathecae.

**Figure 6.**
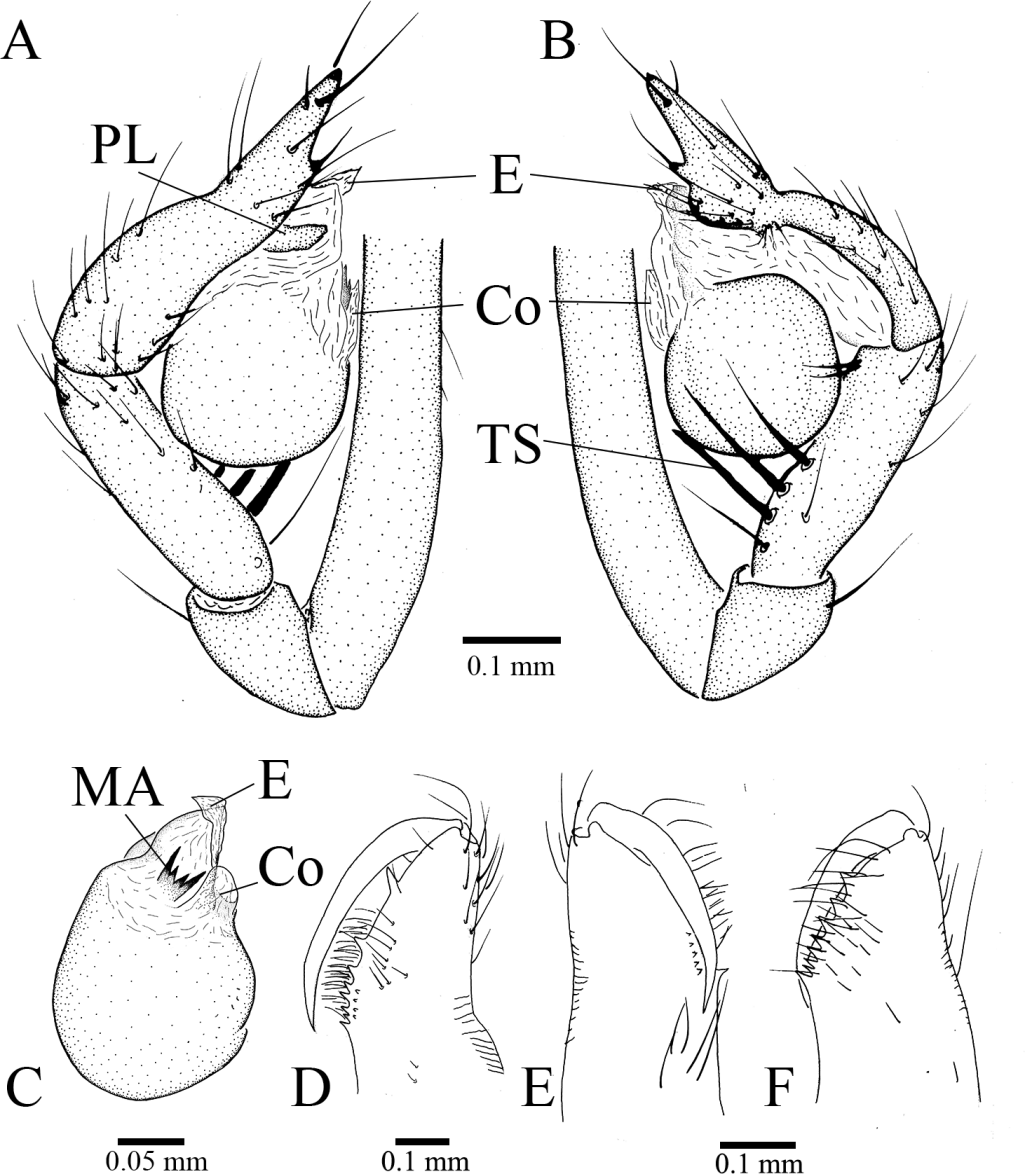
*Leptonetela trispinosa* (Yin et al., 1984), comb. nov., holotype male (**A–D**) and paratype femal (**E, F**) **A** palp, prolateral view **B** palp, retrolateral view **C** palp, ventral view **D** chelicera, retrolateral view **E** right chelicera (because left chelicera is missing), retrolateral view (slightly dorsal) **F** right chelicera, prolateral view. Abbreviations: Co, conductor; E, embolus; MA, median apophysis; PL, prolateral lobe; TS, tibial spur.

The male of *Leptonetela trispinosa* (Yin et al., 1984) comb. nov. is similar to those of *Leptonetela hangzhouensis* (Chen et al., 1984) and *Leptonetela microdonta* (Xu & Song, 1983) in having the median apophysis fork-shaped and a similar arrangement of spines on the retrolateral palpal tibia (compare Figs 4B, 6D with Wang and Li 2011: figs 13B, D, 28B, D), but differs from *L. hangzhouensis* by the shape of the teeth on the median apophysis (the middle two teeth ca half of the lateral two in length in this species vs ca one-third in *L. microdonta*) (compare Fig. 4B with Wang and Li 2011: fig. 13B), and from *L. microdonta* by the number and shape of teeth on the median apophysis (four teeth, the middle two teeth ca half of the lateral two in length in this species vs five teeth, the middle three teeth very small, shorter than one fourth of the lateral two in *L. microdonta*) (compare Figs 4B, 6D with Wang and Li 2011: figs 28B, D). The female of *Leptonetela trispinosa* can be distinguished from that of *Leptonetela microdonta* by the different twisting of the spermathecae (compare Fig. 5E with Wang and Li 2011: fig. 29C, D).

##### Description.

***Holotype*** Male. Body (Fig. 4A) length 1.80, carapace 0.80 long, 0.80 wide, abdomen 1.00 long, 0.73 wide (data from original description by Yin et al. 1984: 364). Carapace yellow brown (Fig. 4A). Six eyes, ALE, and PLE connected to each other by the black bases, PME separated from ALE and PLE. Thoracic median groove short, brown, needle-shaped. Cervical grooves and radial furrows brown, indistinct. Chelicerae tawny, with eight promarginal (teeth gradually becoming smaller and denser from the distal end to the base of chelicera) and four small retromarginal teeth (Fig. 6D). Endites tawny. Labium brown, fused to sternum. Sternum tawny, peltate. Legs yellow; measurements: I 6.80 (2.01, 0.33, 2.33, 0.83, 1.30); II 6.27 (1.70, 0.30, 1.83, 1.43, 1.01); III 5.02 (1.43, 0.23, 1.43, 1.10, 0.83); IV 6.65 (1.93, 0.23, 2.00, 1.39, 1.10) (data from original description by Yin et al. 1984: 364). Abdomen pale brown, oval, lacking distinct patterns (Fig. 4A). Male palp as illustrated in Figs 4B–D, 6A–C. Femur without strong spines. Patella with a small spine dorsally. Trichobothria not found on the dorsal tibia, although they are usually present in the other congeneric species; it is very possible that trichobothria have detached from the body and been lost. Tibia with one seta and five spines retrolaterally (three very strong spines in longitudinal row, other two near distal end of tibia obviously shorter and thinner). Tarsus slightly sunken and contracted at middle resulting in formation of earlobe-shaped process distally (Fig. 4D); one distal spine, one ventral long spine, one long retrolateral spine, and one long prolateral spines present on distal half of tarsus (Figs 4C, D, 6A, B). Palpal bulb oval, smooth. Conductor lamellar, membranous, and slightly wide. Embolus membranous, broad, with the distal part slightly curled towards base (Fig. 6C). Median apophysis fork-shaped, with four teeth, lateral two strong and middle two smaller (Figs 4B, 6C). Prolateral lobe medium-sized, elliptical (Fig. 6A).

***Paratype*.** Female. Similar to male in general features and body size, but coloration paler (Fig. 5A–D). Body length 2.17, carapace 0.90 long, 0.73 wide, abdomen 1.27 long, 0.87 wide (data from original description by Yin et al. 1984: 364). Chelicerae tawny, with eight promarginal and five small retromarginal teeth (Fig. 6E, F). Leg measurements: I 7.08 (2.00, 0.26, 2.13, 1.69, 1.00); II 5.55 (1.60, 0.20, 1.69, 1.26, 0.80); III 4.62 (1.20, 0.20, 1.33, 1.20, 0.69); IV 5.86 (1.73, 0.20, 1.80, 1.26, 0.87) (data from original description by Yin et al. 1984: 366). Genital area densely covered with long hairs. Atrium subtriangular, much wider than long. Internal genitalia consists of paired spermathecae and sperm ducts. Spermathecae highly twisted, with distal ends separated slightly far from each other, and also more strongly sclerotized than proximal part (Fig. 5E).

##### Distribution.

Only known from the type locality, Hunan, China (Fig. 9).

##### Note.

Because of the poor quality of the images in all available references to the female of *L. hangzhouensis* the females of these two species cannot be compared.

#### Leptonetela unispinosa

Taxon classificationAnimaliaAraneaeLeptonetidae

(Yin, Wang & Wang, 1984) comb. nov.

71982CF3-4AE2-5FF2-9EBE-EA6A353185FA

Figures 7 , 8

Leptoneta unispinosa Yin et al. 1984: 368, fig. 3a–d (♂); Song 1987: 107, fig. 70 (♂); Song et al. 1999: 51, fig. 21P–Q (♂, reproduction of the original figure); Yin et al. 2012: 159, fig. 28a–d (♂).

##### Material examined.

***Holotype*** ♂ (HNU, Lept-*Leptonetela*-0002-001): **China**, Hunan Province, Changsha City, Mountain Yuelu, XI.1980, Zhitong Wang leg (information on the label of the type) [Mountain Yuelu: 112°58'N, 28°12'E].

##### Diagnosis.

**Figure 7.**
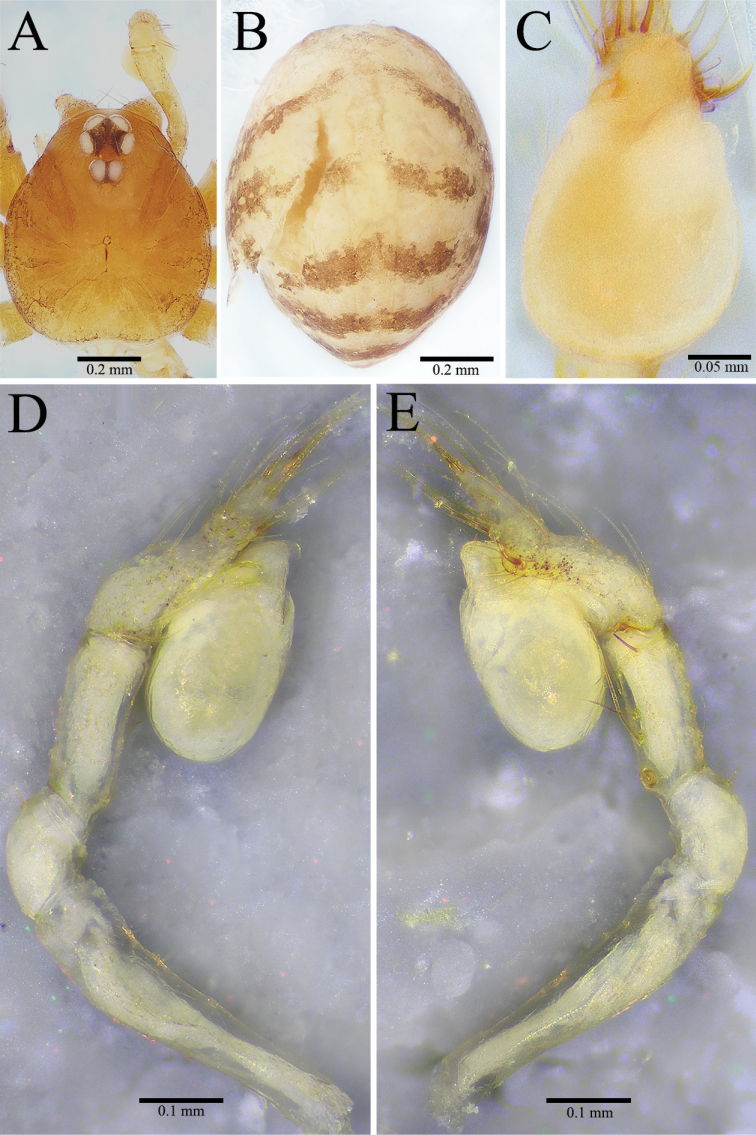
*Leptonetela unispinosa* (Yin et al., 1984), comb. nov., holotype male **A** carapace, dorsal view **B** abdomen, dorsal view **C** palpal bulb, ventral view **D** palp, prolateral view **E** palp, retrolateral view.

**Figure 8.**
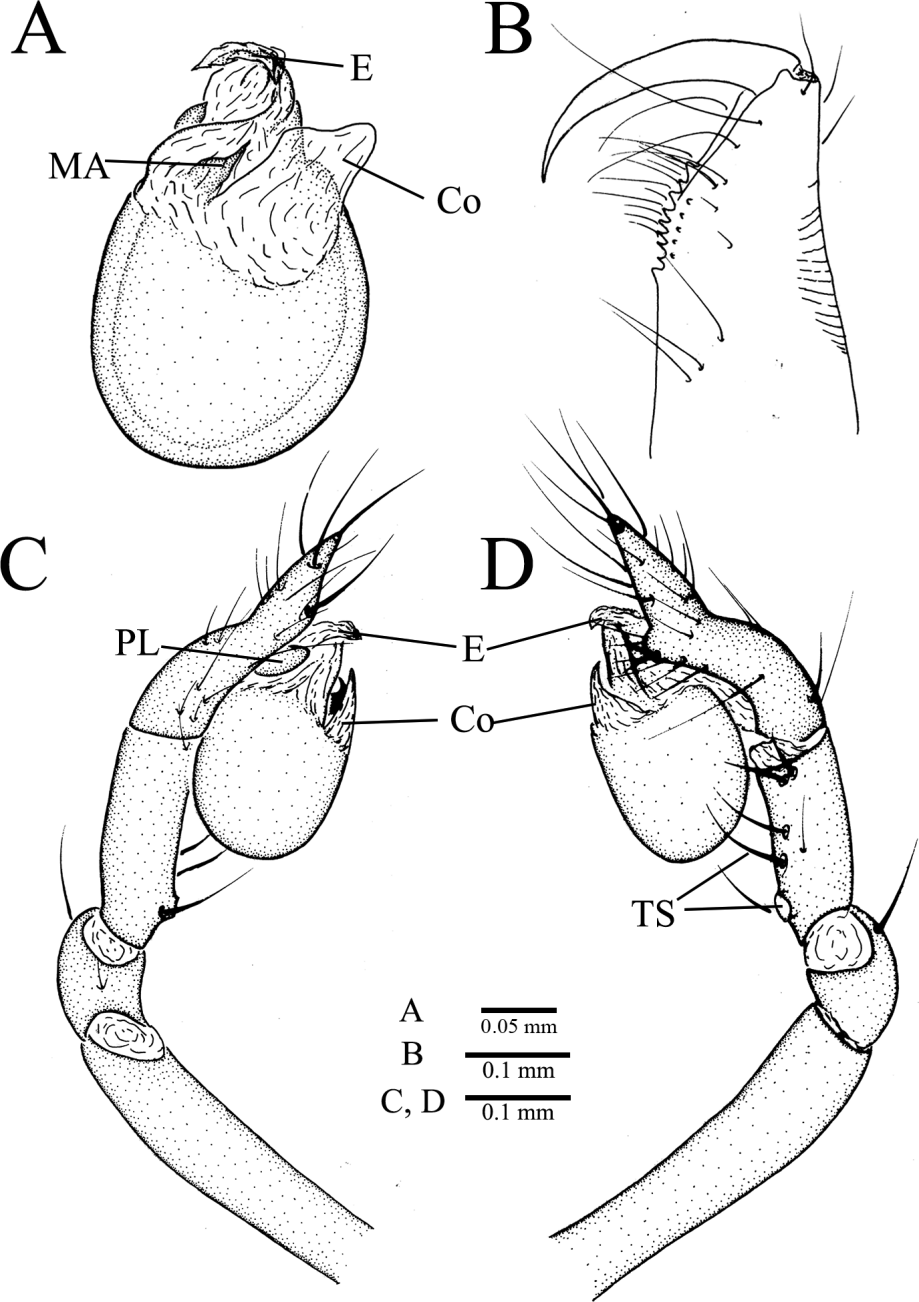
*Leptonetela unispinosa* (Yin et al., 1984), comb. nov., holotype male **A** palpal bulb, ventral view **B** chelicera, retrolateral view **C** palp, prolateral view **D** palp, retrolateral view. Abbreviations: Co, conductor; E, embolus; MA, median apophysis; PL, prolateral lobe; TS, tibial spur.

The male of *Leptonetela unispinosa* (Yin et al., 1984), comb. nov. is similar to that of *Leptonetela quinquespinata* (Chen & Zhu, 2008) in having an A-shaped median apophysis and the embolus curved distally (compare Fig. 8A with Wang and Li 2011: fig. 47D), but differs by the number of eyes (six eyes in this species vs eyes completely absent in *Leptonetela quinquespinata*) and the arrangement of spines on the retrolateral tibia (five spines including three in a longitudinal row and two in a transverse line in this species vs six spines almost in a longitudinal row in *Leptonetela quinquespinata*) (compare Figs 7A, 8D with Wang and Li 2011: fig. 44A, D).

##### Description.

***Holotype*.** Male. Body (Fig. 7A, B) length 1.73, carapace 0.83 long, 0.66 wide, abdomen 1.00 long, 0.66 wide (data from original description by Yin et al. 1984: 367). Carapace brown (Fig. 7A). Six eyes, ALE, and PLE connected to each other by black bases, PME separated from ALE and PLE. Thoracic median groove short, brown, needle-shaped; single shallow pit with brown margin in front of thoracic median groove. Cervical grooves and radial furrows deep brown, indistinct. Chelicerae brown, with nine promarginal and five small retromarginal teeth (all teeth in the same row almost equal in size) (Fig. 8B). Endites brown. Labium deep brown, fused to sternum. Sternum brown, peltate. Legs brown; measurements: I (1.20, 0.26, 1.23, missing, missing); II 3.37 (0.83, 0.24, 0.90, 0.80, 0.60); III 3.00 (0.81, 0.23, 0.73, 0.73, 0.50); IV 4.47 (1.41, 0.24, 1.16, 1.00, 0.66) (data from original description by Yin et al. 1984: 367). Abdomen pale brown, ovoid, with five broad, reddish brown bands dorsally (Fig. 7B).

Male palp as illustrated in Figs 7C–E, 8A, C, D. Femur without any strong spines. Patella with dorsal spine distally. Trichobothria not to be found on dorsal tibia, although usually present in other congenerics (it is very possible that trichobothria were broken off body and lost). Tibia with one long thin prolateral spine basally and five retrolateral spines (three spines arranged in longitudinal row along tibia, first one near basal end especially strong; other two arranged in transverse line along distal margin of tibia). Tarsus sunken and contracted slightly at middle position resulting in forming earlobe-shaped process distally (Fig. 8D). One distal short spine, one ventral long spine, one long retrolateral spine, and one long prolateral spine present on distal half of tarsus (Figs 7D, E, 8C, D). Palpal bulb oval, smooth. Conductor lamellar, membranous, slightly wide. Embolus membranous, slightly twisted towards the prolateral side. Median apophysis A-shaped (Figs 7C, 8A). Prolateral lobe medium-sized, elliptical (Fig. 8C).

##### Distribution.

**Figure 9.**
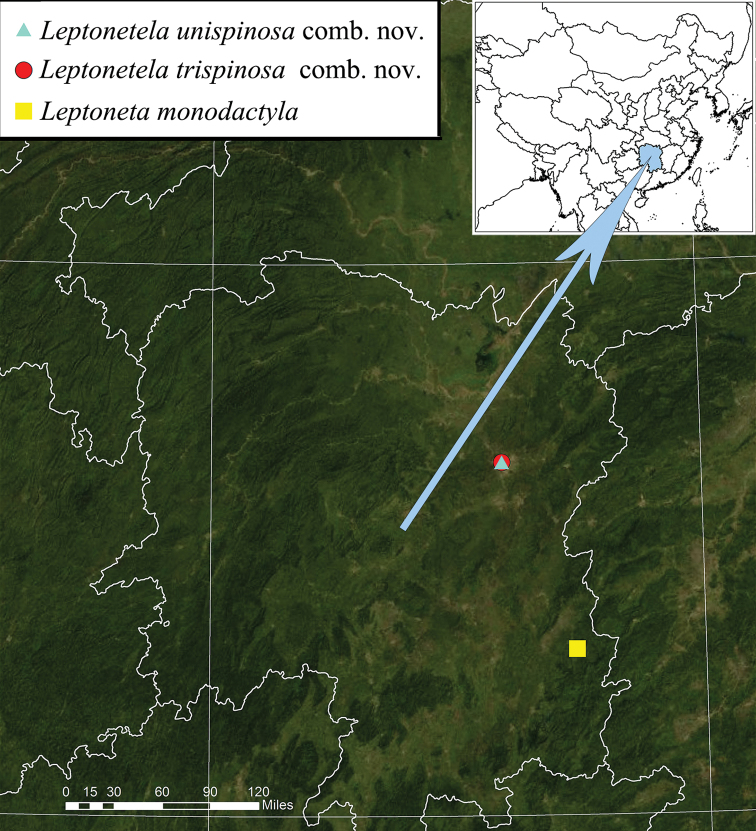
Locality records for *Leptonetela trispinosa* comb. nov., *Leptonetela unispinosa* comb. nov. and *Leptoneta monodactyla*.

Only known from the type locality, Hunan, China (Fig. 9).

## Discussion

The family Leptonetidae comprises 353 species belonging to 21 genera worldwide. Only three genera, *Leptoneta*, *Leptonetela*, and the monotypic genus, *Rhyssoleptoneta* Tong & Li, 2007, are distributed in China (WSC 2020).

Chinese *Leptoneta* species have diverse morphological characteristics of the male palp and Tong and Li (2008) thought that they should probably be included in one or more new genera. Tong and Li divided Chinese *Leptoneta* species (excluding *Leptoneta arquata* Song & Kim, 1991, a species for which only the female known) into four species groups, *Leptoneta maculosa* group, *Leptoneta huanglongensis* group, *Leptoneta microdonta* group, and *Leptoneta miaoshiensis* group. The *Leptoneta microdonta* group consisted of six species and is characterized by the presence of strong spines ventrally on the male palpal tibia (Tong and Li 2008). Three species of the *Leptoneta microdonta* group have been transferred to the genus *Leptonetela* by Wang and Li (2011) and two species are being transferred in the present study. Judging from the characteristics of the male palpal tibia and femur shown in the figures of Chen et al. (2000), we think that *Leptoneta xui* Chen, Gao & Zhu, 2000, the only one remaining in the *L. microdonta* group, should also be a member of the genus *Leptonetela* and that the *Leptoneta microdonta* group should be dropped entirely.

The genus *Leptonetela* is mainly distributed in China. Nighty-eight species (including two new combinations in the current study) are described from China and only 12 from regions outside China including nine from Greece, one from Vietnam, one from Turkey and one from the Caucasus.

The quick increase of the number of Chinese *Leptonetela* species is mainly due to two excellent studies: Wang and Li (2011) and Wang et al. (2017). Twenty-seven and 46 new species were reported by Wang and Li (2011) and Wang et al. (2017), respectively. With three exceptions (*Leptonetela pungitia* Wang & Li, 2011; *Leptonetela trispinosa*; *Leptonetela unispinosa*), nearly all Chinese *Leptonetela* species are endemic to either a single cave or a cave system (Wang et al. 2017; He et al. 2019; WSC 2020). Study of additional caves in China may result in the discovery of more undescribed cave-associated *Leptonetela* species, but this still needs to be confirmed by future collecting.

## Supplementary Material

XML Treatment for Leptoneta

XML Treatment for Leptoneta monodactyla

XML Treatment for Leptonetela

XML Treatment for Leptonetela trispinosa

XML Treatment for Leptonetela unispinosa
